# Cholesterol and its association with muscle weakness in critical illness

**DOI:** 10.1186/s13054-021-03722-2

**Published:** 2021-08-17

**Authors:** Daniel A. Hofmaenner, Anna Kleyman, Mervyn Singer

**Affiliations:** Bloomsbury Institute of Intensive Care Medicine, Division of Medicine, University College London, London, WC1E 6BT UK

Dear Editor,

With great interest we read and appreciated the recently published article by Goossens and colleagues [1]. They offer some important insights into the phenomenon of muscle weakness in critical illness and suggest a contributory role of low cholesterol and potential utility from exogenous administration of 3-hydroxybutyrate. It has long been recognized that low serum cholesterol is commonplace in sepsis and an important prognosticator of poor outcomes [2, 3]. They reported that 3-hydroxybutyrate (3-HB) increased plasma cholesterol levels in septic mice with normalization of plasma mevalonate and increased expression of genes encoding cholesterol synthesis in muscle.

We would like to raise some points of interest that merit discussion. First, the authors measured serum cholesterol levels in patients on ICU admission and day 3, whereas muscle strength was assessed at day 8. What was the rationale for the choice of these timepoints? Ideally, comparison of muscle strength should be made against contemporaneous cholesterol levels to reduce risks of confounding and to support causality arguments.

Second, did the authors consider measuring endogenous production of 3-HB and plasma levels in their septic animals? Endogenous hydroxybutyrate levels are altered in sepsis due to a shift towards fat metabolism and increased ketone body production [4]. Understanding of the pharmacokinetics/pharmacodynamics of exogenous 3-HB administration would aid interpretation of its impact.

Third, the authors present the fate of supplemented 3-HB as cholesterogenic or tricarboxylic acid cycle (TCA cycle) substrate (Figure 3). A complementary pathway worth considering is fatty acid synthesis which also relies upon acetyl-CoA as a basic substrate. Again, temporal measurement across the catabolic and recovery phases of sepsis would be informative.

Fourth, to our knowledge, the liver produces ketone bodies but cannot directly metabolize them due to a lack of ketoacyl-CoA transferase. The observed elevation in mevalonate within the liver after exogenous 3-HB administration may relate to indirect metabolic pathways, e.g. via fatty acids transported back to the liver.

## Data Availability

Not applicable.
