# Supplementary material for: Cholesterol and its association with muscle weakness in critical illness
Source: Crit Care. 2021 Aug 17;25:296. doi: 10.1186/s13054-021-03722-2 (PMC8371860; doi:10.1186/s13054-021-03722-2)
Supplement: Related texts

# Reply: Authors’ response

**Authors:** Lies Langouche, Greet Van den Berghe

**Affiliations:** grid.5596.f 0000 0001 0668 7884 Clinical Division and Laboratory of Intensive Care Medicine, Department of Cellular and Molecular Medicine, KU Leuven, 3000 Leuven, Belgium

## Reply to letter by Hofmaenner D.

We thank Hofmaenner D. *et al*. for the interest in our work on the role of cholesterol in ICU acquired weakness and the impact thereof on exogenous 3-hydroxybutyrate (3HB) and for raising several interesting points [1].

The first point addressed the difference in timing between measurement of serum cholesterol and of muscle strength in the human study (EPaNIC RCT). In this study, the impact of omitting parenteral nutrition for one week in ICU on ICU acquired weakness was assessed in a matched subgroup of 600 patients on post-randomization day 8 [5]. Muscle strength assessment was performed in ICU for long-stay patients, and on the regular ward for short-stayers. Blood was only sampled during ICU stay, and thus no later samples were available for short-stayers. For the current analysis of a potential association between plasma cholesterol and ICU acquired weakness, we therefore quantified plasma cholesterol in all patients on one timepoint, day 3 in ICU, which preceded the muscle strength assessment, as selection bias would otherwise occur at a later time point.

Second, the authors asked whether data on endogenous production of 3HB and plasma concentrations were available for the septic mice. Indeed, as mentioned by Hofmaenner D. *et al*., endogenous production of 3HB is increased in the acute phase of critical illness, but this is abolished as soon as parenteral nutrition is initiated, as we have previously shown both in the septic mouse model [6], and in human patients [7, 8]. We agree that further insight in the pharmacokinetics/pharmacodynamics of exogenous 3HB administration would be informative when considering its further investigation in human ICU patients.

The third and fourth points addressed the metabolic fate of the exogenous 3HB and whether use as substrate for fatty acid synthesis could be involved. Indeed, 3HB can in theory be converted into fatty acids. In the mouse model, 3HB supplementation has previously shown not to alter plasma fatty acid concentrations or triglyceride content in muscle or liver [6], whereas it was found to increase plasma triglyceride concentrations [6]. Indirectly, it is also possible that conversion of 3HB to fatty acids or mevalonate in non-hepatic tissues may have occurred which could then be further metabolized in the liver.

We totally agree with Hofmaenner D. *et al*. that more research is needed to better understand the role of cholesterol in ICU acquired weakness and to assess the clinical potential of 3HB supplementation in human ICU patients.
